# Mortality, Rehospitalisation and Violent Crime in Forensic Psychiatric Patients Discharged from Hospital: Rates and Risk Factors

**DOI:** 10.1371/journal.pone.0155906

**Published:** 2016-05-19

**Authors:** Seena Fazel, Achim Wolf, Zuzanna Fimińska, Henrik Larsson

**Affiliations:** 1 Department of Psychiatry, Oxford University, Warneford Hospital, Oxford, OX3 7JX, United Kingdom; 2 Department of Medical Epidemiology and Biostatistics, Karolinska Institute, Stockholm, Sweden; Shinshu University School of Medicine, JAPAN

## Abstract

**Objectives:**

To determine rates and risk factors for adverse outcomes in patients discharged from forensic psychiatric services.

**Method:**

We conducted a historical cohort study of all 6,520 psychiatric patients discharged from forensic psychiatric hospitals between 1973 and 2009 in Sweden. We calculated hazard ratios for mortality, rehospitalisation, and violent crime using Cox regression to investigate the effect of different psychiatric diagnoses and two comorbidities (personality or substance use disorder) on outcomes.

**Results:**

Over mean follow-up of 15.6 years, 30% of patients died (n = 1,949) after discharge with an average age at death of 52 years. Over two-thirds were rehospitalised (n = 4,472, 69%), and 40% violently offended after discharge (n = 2,613) with a mean time to violent crime of 4.2 years. The association between psychiatric diagnosis and outcome varied—substance use disorder as a primary diagnosis was associated with highest risk of mortality and rehospitalisation, and personality disorder was linked with the highest risk of violent offending. Furthermore comorbid substance use disorder typically increased risk of adverse outcomes.

**Conclusion:**

Violent offending, premature mortality and rehospitalisation are prevalent in patients discharged from forensic psychiatric hospitals. Individualised treatment plans for such patients should take into account primary and comorbid psychiatric diagnoses.

## Introduction

Secure hospitals typically treat individuals with severe mental disorders who are at increased risk of violence. Also known as forensic psychiatric institutions, they admit either prisoners whom prison medical services are not able to manage appropriately, individuals who have been admitted from court following a serious criminal offence, or psychiatric patients who cannot be managed in general wards.[1] Over the last twenty years, there has been a large increase in the number of secure psychiatric hospital beds in many high-income countries.[2, 3] Forensic patients cost €190,000 annually per patient in low secure institutions to €340,000 at high secure hospitals.[1] In England and Wales, the overall budget of over €1.2 billion[4] is equivalent to 19% of the overall mental health budget, and represents its largest single component, while serving around 1% of all patients who make contact with psychiatric services.[5] Equivalent information is not available in other countries but there are higher rates of forensic psychiatric beds in several Western European countries than the UK, such as the Netherlands, Germany, Austria and Denmark, with well-developed services.[3] In Alberta, Canada, the estimated cost of a not-criminally-responsible forensic case is €190,000 per year.[6] In Sweden, approximately 1000 patients (around 10 per 100,000 population) are inpatients in forensic psychiatric hospitals at any given time, with an estimated annual cost per patient of €250,000 in 2014 prices.[7]

Outcomes after discharge have been investigated in some countries. Although short-term reconviction rates appear to be low, in the longer term, absolute rates of adverse outcomes are high with up to half being reconvicted,[1] mortality rates in two studies of 18%[8] and 23%,[9] and increased readmission rates.[10] Few studies, however, have investigated multiple adverse outcomes in forensic psychiatric patients. For example, two English population-based studies investigated only offending outcomes and in patients from medium secure units (hence excluding low and high secure patients).[11, 12] Various studies have investigated specifically high secure patients,[13–15] or other selected population such as insanity acquitees.[16, 17]

Further, with such high rates, information on risk factors is necessary to target interventions. However, little is known about modifiable determinants of these outcomes and whether they are shared across outcomes. Previous work has mostly focused on unmodifiable demographic factors, such as age, gender, and previous offences which increase risk of reoffending.[18] The one report that did investigate multiple adverse outcomes in 554 discharges from one medium secure unit in England presented rates but did not study risk factors.[19] In particular, there is uncertainty whether psychiatric diagnoses are associated with recidivism. The studies that have investigated diagnostic risk factors have restricted their outcome to offending, one of which was based on 100 forensic patients and did not find any significant associations, likely due to the small sample size,[20] and another of 1344 patients in medium security found associations between violent offending and personality disorder but was underpowered to report on other mental disorders, including alcohol and drug dependence.[12] A Canadian study based on 1784 forensic patients found increased risk of reoffending in those with comorbid personality and substance use disorders, and non significant increases in those with psychotic spectrum, and mood spectrum disorders.[21] Similarly, a study on conditional release found that having a one of these comorbidities was related to the revocation of release.[22] General population studies are not possible to generalise to forensic patients but suggest some gradient in risk with substance use increasing risk substantially,[23] more than schizophrenia,[24, 25] bipolar disorder, and personality disorder.[26] As for secondary or comorbid diagnoses, the evidence is considerably weaker, and although some studies in general population samples have reported that comorbid substance use[27, 28] and personality disorder[29] are potentially linked with poor outcomes, the findings are not consistent, with less information in forensic samples. In spite of these limitations, risk assessment tools, such as the HCR-20, which are extensively used to assist in managing those at high risk of reconviction, include clinical items such as major mental illness, personality disorder, and substance use disorder. Similar limitations exist in the evidence base regarding modifiable risk factors for mortality and rehospitalisation outcomes. Substance use disorder may be associated with premature mortality in psychiatric patients,[30, 31] and age and substance use with rehospitalisation in schizophrenia,[32] psychosis,[33] and bipolar disorder.[34] However, it is not known if these findings are relevant to forensic psychiatric samples.

In a large national cohort of all 6,505 patients discharged from secure hospitals in Sweden, we investigated rates and risk factors for mortality, rehospitalisation, and violent crime. By linking Swedish national registers, which provide information on primary and comorbid (personality or substance use disorder) diagnoses, we examined whether specific psychiatric diagnoses and these two comorbidities were associated with adverse outcomes. We hypothesised no differences by primary diagnosis, but in keeping with work in general psychiatric settings,[23, 25] we anticipated that comorbidity would increase risk of mortality, rehospitalisation, and violent crime.

## Methods

We linked three high quality longitudinal Swedish population registers: the Patient Register, the Cause-of-Death Register, and Crime Register. Swedish citizens have a unique identification number that can be used to link data across registers. The Patient Register contains information on diagnoses of all individuals who are admitted to any general, psychiatric, or secure hospital for assessment or treatment. All patients are given clinical diagnosis on discharge according to ICD-9 (until 1996) and ICD-10 (from 1997) (International Classification of Diseases, 9^th^ and 10^th^ revisions). This register is reported to be valid and reliable for a range of psychiatric diagnoses,[35] including schizophrenia, other psychoses, bipolar disorder,[36] and personality disorders, and also for comorbid substance use.[37] Overall, the positive predictive value has been reported to be 85–95% for most diagnoses.[35] The Crime Register includes conviction data for people aged 15 (the age of criminal responsibility) and older.[38]

Data was first extracted on 7,948 individuals who were admitted to a forensic psychiatric hospital between 1973 and 2009 (the entire period for which register data was available). The data were checked for duplicates, but none were detected. Individuals who died during their hospital stay, or were transferred to another psychiatric institution were excluded from the analysis, giving the total sample of 6,520. Ethics approval was obtained from the Regional Ethics Committee at Karolinska Institutet (2009/939-31/5). Data were merged and anonymised by an independent government agency (Statistics Sweden), and the code linking the personal identification numbers to the new case numbers was destroyed immediately after merging. Therefore, informed consent was not required.

In Sweden, similar to most Western countries, psychiatric assessments can be requested to inform decisions about sentencing and transfer to hospital. These assessments are routinely requested by the court in those with a known history of psychiatric disorder, or whose offence characteristics or behaviour in police custody has raised mental health concerns.[39] These assessments are made by specialist psychiatrists (state-certified general adult with a specialty in forensic psychiatry) psychiatrists who review legal and medical records, and conduct a clinical interview in which standardised diagnoses are recorded according to the Diagnostic and Statistical Manual of Mental Disorders (DSM-III-R until 1996, and subsequently DSM-IV). This can be as an outpatient (a ‘minor’ forensic psychiatric examination), but in more serious cases, the courts order an inpatient assessment (a ‘major’ forensic psychiatric examination) that normally takes 3–4 weeks, and leads to a multidisciplinary report that addresses whether the alleged crime was committed under the influence of a severe mental disorder. The report is then used as a basis for the court to decide whether the individual receives ongoing forensic psychiatric care, and in almost all cases, the court follows the recommendation of the forensic psychiatric examination. High, medium and low security wards are available. In 2007, 8% of the persons detained in forensic psychiatric units are for murder/manslaughters, 27% for assault, 3% for sexual crimes, 13% for arson, and the main diagnoses were reported to be psychoses (68%), personality disorder (13%), behavioural disorders (11%), and drug-related disorders (5%).[40] Around two thirds have ‘special court supervision’ that means that discharge decisions need approval by the county administrative court.[40]

### Risk factors

Information on demographic factors was collected from the Patient Register, which includes data on age, gender, and place of birth. Data on the most recent offence (also known as the index offence) was gathered from the Crime Register, and we used a standard system to classify crimes into six main categories.[37, 41] From the Patient Register, the following psychiatric diagnoses on discharge were investigated: schizophrenia and related disorders (ICD-8: 295, 297–299; ICD-9: 295, 297–299 excl. 299A; ICD-10: F20-F29), bipolar disorder (296 excl. 296.2; 296 excl. 296D; F30-F31), depression (296.2, 300.4; 296D, 300E, 311; F32-F34.1), anxiety disorders (300 excl. 300.4; 300 excl. 300E; F40-F42, F45), substance use (291, 294.3, 303–304; 291–292, 303–305; F10-F19), personality disorders (301; 301; F60-F62), learning disability (310–315; 317–319; F70-F79), developmental disorders (306.0, 306.1, 306.3; 299, 315; F80-F89) and organic disorder (290, 292–294 excl. 294.3, 309; 290, 293–294; F00-F09).

### Outcomes

Data on deaths was provided by the Cause of Death register, which reports the date and cause of death, as well as the national registration number, allowing linkage with other registers. Death certificates are used as the source of information for the cause-of-death register. Causes of deaths are classified according to the ICD codes.[42]

Information on rehospitalisation was extracted using the Patient Register, which provides dates of hospital admission and discharge, as well as the main discharge diagnosis (and secondary diagnosis, where applicable) according to ICD.[43] Rehospitalisation was defined as hospitalisation in any psychiatric inpatient hospital. Violent crime was defined as homicide, assault, robbery, arson, any sexual offence (rape, sexual coercion, child molestation, indecent exposure, or sexual harassment), illegal threats or intimidation (hence burglary and other property, traffic, and drug offences were excluded).[25] Conviction data were used because the criminal code in Sweden determines that individuals are convicted as guilty regardless of mental illness. Therefore, it includes those who are found not guilty by reason of insanity (who would be acquitted in other countries), individuals transferred to forensic hospitals, and those receiving cautions and fines. In addition, plea bargaining is not permitted in Sweden; conviction data accurately reflect the extent of officially resolved criminality. The crime register has excellent coverage; only 0.05% of crimes had incomplete personal identification numbers in 1988–2000.[41]

### Statistical analyses

Cox regression models were constructed separately for each diagnosis and outcome. Three types of analysis were conducted. First, hazard ratios for primary diagnoses were calculated relative to schizophrenia and related disorders, the latter constituting the largest diagnostic group and hence acted as reference (n = 2,188; 33.6%). Hazard ratios were cumulatively adjusted for age (at first discharge) and sex, previous violent offence, index violent crime, and secondary diagnoses of substance use disorder and personality disorder. Thus the hazard ratios of each diagnosis relative to schizophrenia-spectrum disorders are presented for each adverse outcome. The variables entered into the model were determined a priori based on previous research on recidivism in mentally disordered offenders.[39] The analysis was performed using the command ‘stcox’ in Stata 12.1 by entering time at risk for a particular outcome, the outcome as a binary variable, and covariates.

Second, we stratified the sample according to two comorbidities previously found to be associated with violence (personality disorder and substance use disorder),[28] and explored the same three adverse outcomes (mortality, psychiatric readmission, and violent offending). To do so, patients with a particular diagnosis were selected, and, following adjustment for age and gender, secondary diagnoses were entered as covariates, allowing us to compare patients with a given comorbidity within a particular diagnostic group. Finally, we examined the relationship between length of stay and adverse outcomes, with and without adjustment for risk factors.

All analyses were performed in Stata 12.1.

## Results

We identified 6,505 patients discharged from forensic psychiatric hospitals over 1973–2009 who were predominantly male (89.2%), with a mean age at discharge of 36.6 years (S.D. 11.5, range 15.7–83.4). Of these, 1,609 (24.7%) were non-Swedish citizens, and 6,244 (96.8%) had been admitted to hospital following conviction of a violent offence. The five largest diagnostic categories were: schizophrenia and related disorders (33.6%), bipolar disorder (4.9%), depression (4.0%), substance use (17.1%), personality disorders (25.8%) (Table 1).

**Table 1:** Clinical and criminal characteristics of 6,520 adults released from forensic psychiatry between 1973 and 2009.

		n (%)
**Primary diagnosis at first discharge**	Schizophrenia and other psychoses	2,188 (33.6%)
	Bipolar disorder	321 (4.9%)
	Depression	258 (4.0%)
	Anxiety	202 (3.1%)
	Substance use	1,114 (17.1%)
	Personality disorders	1,680 (25.8%)
	Learning disability	132 (2.0%)
	Developmental disorder	115 (1.8%)
	Organic disorder	149 (2.3%)
	Other	361 (5.6%)
**Secondary diagnosis at first discharge**	Substance abuse	1,458 (22.4%)
	Personality disorder	638 (9.8%)
Type of index offence*	Homicide and attempted homicide	792 (12.1%)
	Aggravated assault	1,595 (24.5%)
	Common assault	707 (10.8%)
	Assaulting an officer	753 (11.6%)
	Sexual offences	616 (9.4%)
	Robbery	364 (5.6%)
	Arson	661 (10.1%)
	Threats and harassment	1,893 (29.0%)
	Non-violent crime only	1,330 (20.4%)

*Some cases committed more than one type of crime at index offence.

### Mortality

Average time at risk for mortality was 15.6 years (S.D. 10.2); during the follow-up time there were 1,949 deaths (29.9%), giving the overall rate of 1,916 deaths/100,000 person-years. In our cohort, 443 (6.8%) people died within five years of discharge; 839 (12.9%) died within ten years of discharge. Average age at death was 52.2 years (S.D. 12.6). Of the 1,949 deaths, 35 (1.8%) were recorded as homicides, 443 (22.7%) as suicides, and 277 (14.2%) as accidental deaths.

**Fig 1:**
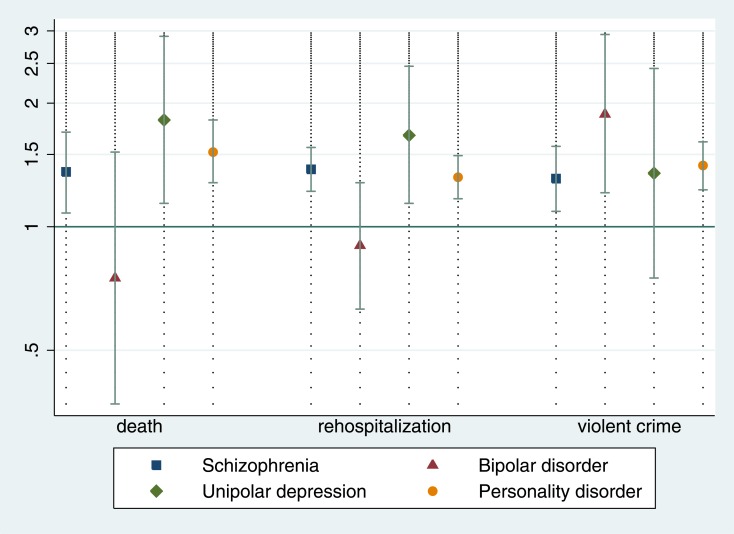
The effect of comorbid substance use disorders on the risk of death, rehospitalisation, and violent offending in a cohort of forensic patients. Hazard ratios are age and gender-adjusted. Hazard ratio of 1 means no effect of comorbid substance use disorders.

**Fig 2:**
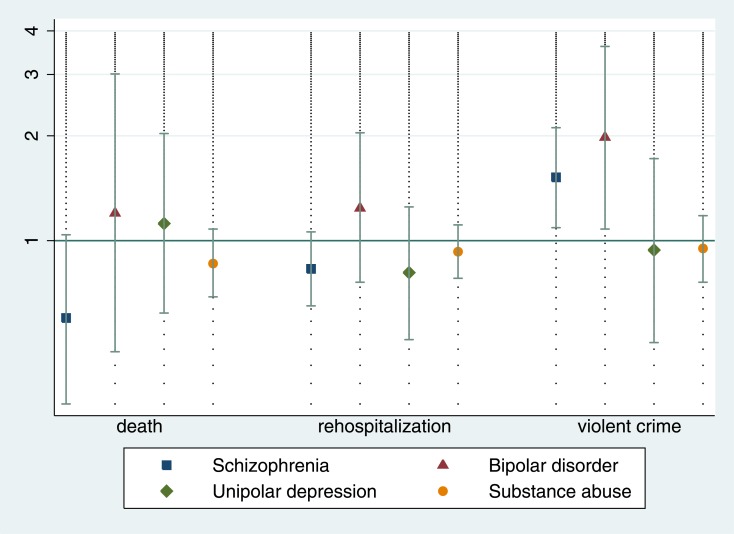
The effect of comorbid personality disorder on the risk of death, rehospitalisation, and violent offending in a cohort of forensic patients. Hazard ratios are age and gender-adjusted. Hazard ratio of 1 means no effect of comorbid personality disorder.

Compared with schizophrenia and related disorders (which was the reference diagnostic category), primary diagnoses of substance use disorder increased hazards of mortality (Table 2). When primary psychiatric diagnoses were comorbid with substance use disorder, mortality risk was increased in all groups apart from bipolar disorder (Fig 1). No clear associations were found for comorbid personality disorder (Fig 2).

**Table 2:** Hazard ratios (95% Confidence intervals) of psychiatric risk factors for mortality in a cohort of forensic patients.

Primary diagnosis	Age & sex	+ previous violent crime	+ secondary PD and SUD
Schizophrenia-spectrum	1.00 (ref)	1.00 (ref)	1.00 (ref)
Bipolar disorder	0.96 (0.76–1.23)	0.95 (0.74–1.21)	0.95 (0.75–1.22)
Unipolar depression	1.21 (0.97–1.52)	1.26 (1.00–1.58)	1.28 (1.02–1.62)
Substance use disorder	1.79 (1.58–2.02)	1.73 (1.53–1.96)	n/a
Personality disorder	1.10 (0.97–1.25)	1.11 (0.98–1.26)	n/a

Adjusted cumulatively for age and sex, previous violent crime (index or previous), secondary substance use disorder (SUD) and secondary personality disorder (PD). All values are presented relative to patients with schizophrenia-spectrum disorders.

### Rehospitalisation

Average time at risk for rehospitalisation was 5.3 years (S.D 8.2). During the follow-up, there were 4,472 (68.6%) rehospitalisations, equivalent to 12,941 readmissions per 100,000 person-years.

Schizophrenia and related disorders were associated with increased hazards of rehospitalisation compared to other depression and personality disorder, but associated with lower hazards compared to bipolar and substance use disorder (Table 3). Comorbid substance use disorder increased risk of rehospitalisation in all primary diagnoses investigated apart from bipolar disorder, with no significant associations were found for comorbid personality disorder.

**Table 3:** Hazard ratios (95% Confidence intervals) of psychiatric risk factors for rehospitalisation in a cohort of forensic patients.

Primary diagnosis	Age & sex	+ previous violent crime	+ secondary PD and SUD
Schizophrenia-spectrum	1.00 (ref)	1.00 (ref)	1.00 (ref)
Bipolar disorder	1.22 (1.06–1.40)	1.20 (1.05–1.38)	1.22 (1.06–1.40)
Unipolar depression	0.75 (0.63–0.88)	0.78 (0.66–0.93)	0.78 (0.66–0.93)
Substance use disorder	1.30 (1.20–1.41)	1.25 (1.15–1.36)	n/a
Personality disorder	0.78 (0.72–0.85)	0.78 (0.72–0.84)	n/a

Adjusted cumulatively for age and sex, previous violent offence, index violent offence, secondary substance use disorder (SUD) and secondary personality disorder (PD). All hazards are presented relative to patients with schizophrenia-spectrum disorders.

### Post discharge violent offending

Over a mean follow-up of 9.4 years (S.D. 9.3), there were 2,613 (40.1%) new violent offences, equivalent to 4,263 offences/100,000 person-years. In our cohort, 1,863 (28.6%) committed a new violent offence within five years of discharge; 2,329 (35.7%) committed a new violent offence within ten years of discharge.

Relative to the reference category of schizophrenia-spectrum disorders, bipolar disorder, substance use disorder, and personality disorder were associated with increased hazards of post-discharge violent offending (Table 4). In addition, comorbidity with substance use disorder and personality disorder typically increased hazards of violent crime after hospital discharge (Figs 1 and 2). Absolute rates of adverse outcomes are presented in S1 and S2 Tables.

**Table 4:** Hazard ratios (95% Confidence intervals) of psychiatric risk factors for violent offending in a cohort of forensic patients.

Primary diagnosis	Age & sex	+ previous violent crime	+ secondary PD and SUD
Schizophrenia-spectrum	1.00 (ref)	1.00 (ref)	1.00 (ref)
Bipolar disorder	1.34 (1.10–1.65)	1.39 (1.13–1.70)	1.40 (1.14–1.72)
Unipolar depression	0.92 (0.72–1.18)	1.05 (0.82–1.35)	1.00 (0.78–1.30)
Substance use disorder	1.67 (1.49–1.87)	1.71 (1.52–1.92)	n/a
Personality disorder	1.73 (1.57–1.92)	1.74 (1.57–1.93)	n/a

Adjusted cumulatively for age and sex, previous violent offence, index violent offence, secondary substance use disorder (SUD) and secondary personality disorder (PD). All hazards are presented relative to patients with schizophrenia-spectrum disorders.

### Length of stay

The median length of stay was 5.1 months (Interquartile Range: 1.7–12.7 months). A longer length of stay was associated with reduced risk of adverse outcome. After adjusting for relevant risk factors, the association remained for risk of rehospitalization and violent crime (Table 5).

**Table 5:** Length of stay and adverse outcomes.

	Hazard Ratio (95% Confidence interval) per extra year in hospital		
	Mortality	Rehospitalization	Violent crime
**Unadjusted**	0.94 (0.90–0.99)	0.88 (0.86–0.91)	0.95 (0.92–0.98)
Adjusted*	0.98 (0.93–1.02)	0.89 (0.87–0.92)	0.92 (0.89–0.96)

*adjusted for age at discharge, sex, previous violent offence, index violent offence, primary diagnosis, secondary substance use disorder, and secondary personality disorder

### Results summary

The effects of psychiatric diagnosis were different across adverse outcomes. Primary substance use carried the most risk for mortality or rehospitalization, while personality disorder was associated with the highest hazards of subsequent violent crime. Compared with schizophrenia-spectrum disorders, bipolar disorder, personality disorder, and substance use increased the hazards of post-discharge violent offending. Similarly, the effect of personality or substance use disorder comorbidity differed by diagnosis and outcome. Comorbid substance use typically increased hazards of adverse outcomes, but there were no significant associations between comorbid personality disorder and mortality and rehospitalisation.

## Discussion

This study aimed to examine the impact of psychiatric diagnosis and comorbidity on the risk of three important outcomes (mortality, readmission, and violent offending) in a cohort of forensic psychiatric patients discharged from hospitals between 1973 and 2009. Although we hypothesised no differences by primary diagnosis, and that personality or substance use disorder comorbidity would increase the risk of all adverse outcomes, the analyses revealed that both primary and secondary diagnoses were important in determining risk. For example, we found that primary and secondary substance use disorder increased risk of mortality, which is in keeping with existing literature.[44–46] In addition, comorbid substance use and personality disorder increased the risk of violent offending, consistent with previous research.[37, 47, 48]

Our principal findings are comparable to other studies in Western countries, which mostly investigate one adverse outcome in isolation in forensic psychiatric patients. We compared our main outcomes with recent systematic reviews. In terms of mortality, we reported a mortality rate of 1,916 per 100,000 person-years compared to 1,538 in a pooled estimate of 35 other studies.[49] For violent reoffending, the rate in this study was also slightly higher at 4,263 vs 3,902 in the systematic review.[49] As for predictors of violent recidivism, a recent overview finds that substance abuse and personality disorder are stronger risk factors than mental illness similar to our findings, but this review included heterogenous populations of psychiatric outpatients and prisoners in addition to discharged forensic psychiatric patients.[18] Although rates of violent crime are lower than comparative populations such as prisoners of similar ages, lengths of stay, and offence types,[50] they remain high compared to non-forensic patient samples, and individuals in the general population.[25] Identifying individuals at high risk remains important despite current approaches being limited in accuracy,[51] authorship effects,[52] and inconsistencies in thresholds for high risk.[53]

A number of clinical implications arise from these findings. First, it suggests that, even if treating comorbid substance use is difficult,[54] forensic psychiatric services should invest greater resources into the assessment and management of these disorders in order to reduce post-discharge mortality[45] and violent offending. Tailored treatments may be necessary as most trials for substance abuse treatment do not include populations comorbid with severe mental illness. Furthermore, regular follow up may be necessary to implement such therapies, and services should be adequately funded to meet these treatment needs. Pharmacological research suggests that clozapine may offer benefits over other antipsychotics,[54] which adds to evidence on their efficacy to reduce violent arrest rates.[55] It should be noted, however, that substance use is unlikely to exist in isolation from other risk factors, and further work should investigate medication adherence[56] and participation in psychosocial treatments as possible mediators of the effects of substance abuse. Motivational interviewing should also be considered. In addition, violent offending can occur in the absence of substance abuse, and consideration of other risk factors is required,[57] including criminal, socio-demographic, and clinical factors previously found to be related to violence.[28] Furthermore innovative ways to target risk factors for mortality may be required considering the patient population, including models of shared care and staff training.[58] Psychoeducation and screening for health parameters without links to other interventions have not been shown to be effective,[59] although improving adherence to guidelines for monitoring physical health is recommended by expert reviews as they target relevant risk factors for cardiovascular mortality.[60] More research into preventing mortality in this patient group is required.

Differing effects of principal psychiatric diagnosis and comorbidity were also relevant to the risk of hospital readmission. In particular, patients with schizophrenia and related disorders were at an increased risk of readmission compared to other diagnostic categories examined. This finding is partly in keeping with one previous study of frequently hospitalised psychiatric patients.[33] Our findings underscore the need for forensic services to follow best practice guidelines to reduce readmission including optimal antipsychotic treatment and psychosocial therapies.[61–63] The role of legally mandated community treatment and equivalent orders,[64] and their relevance for forensic outpatients will need further research. An unexpected finding was that comorbid personality disorder did not increase the risk of readmission, a result not found in general psychiatry.[33] One explanation is that individuals with personality disorders have a higher threshold for readmission, due to more limited treatment options being available, or may be diverted to the criminal justice system.[65]

There were some important limitations. First, we were unable to assess clinical factors beyond primary and secondary diagnoses, such as adherence with medication,[47] social support, service provision, specific symptoms,[28] different types of illegal substance use, sub-threshold substance use problems (such as binge drinking), and personality traits,[66] which may be of particular relevance to offenders with mental illness.[39] Second, we underestimated the true prevalence of violence and antisocial behaviour by using violent convictions as one of the outcomes, but at the same time, these are likely to be the severer types of antisocial behaviour with more consequences for patients, victims, and services. However, this underestimate is unlikely to alter the risk estimates reported because the outcomes are compared within the cohort. Furthermore, using conviction data has the advantage of avoiding the reporting biases associated with self-report and informant questionnaires for crime and potentially allows for international comparisons.[25] In addition, we did not investigate inpatient rates and risk factors[67] of adverse outcomes, which would require more sensitive measures of covariates and outcomes. In particular, violent incidents may not lead to official criminal charges and the lower rates of mortality would require international collaborations or meta-analyses to render estimates stable.

### Conclusion

Serious adverse outcomes after discharge from forensic psychiatric services vary by primary and secondary diagnosis. This suggests different pathways to premature mortality, rehospitalisation, and violent crime in patients discharged from these services, and management that needs to be individualised[68] and tailored to specific psychiatric diagnoses and needs.

## Supporting Information

S1 Table Rates of adverse outcome by diagnostic group, stratified by comorbid substance use disorder (SUD). (DOCX)

S2 Table Rates of adverse outcome by diagnostic group, stratified by comorbid personality disorder (PD). (DOCX)
