# Correction: Mortality, Rehospitalisation and Violent Crime in Forensic Psychiatric Patients Discharged from Hospital: Rates and Risk Factors

**DOI:** 10.1371/journal.pone.0159020

**Published:** 2016-07-05

**Authors:** Seena Fazel, Achim Wolf, Zuzanna Fimińska, Henrik Larsson

There is an omission error in the Methods. After this sentence in the Methods (2nd paragraph, first line): "Data was first extracted on 7,948 individuals who were admitted to a forensic psychiatric hospital between 1973 and 2009 (the entire period for which register data was available)", the following sentence should be added: "As specialist forensic psychiatric hospitals were uncommon in the 1970s and 1980s, our sample includes all patients who were transferred to any psychiatric hospital following a court decision, and thus our sample is not limited to those discharged from secure or forensic hospitals but also those discharged from some general hospitals."
