# Supplementary material for: Mortality, Rehospitalisation and Violent Crime in Forensic Psychiatric Patients Discharged from Hospital: Rates and Risk Factors
Source: PLoS One. 2016 May 19;11(5):e0155906. doi: 10.1371/journal.pone.0155906 (PMC4873227; doi:10.1371/journal.pone.0155906)
Supplement: S1 Table — (DOCX) [file pone.0155906.s001.docx]

**S1 Table.** Rates of adverse outcome by diagnostic group, stratified by comorbid substance use disorder (SUD)

|  | Death | | Rehospitalisation | | Violent crime | |
| --- | --- | --- | --- | --- | --- | --- |
|  | No SUD | SUD | No SUD | SUD | No SUD | SUD |
| Schizophrenia-spectrum | 372 (21.1%) | 97 (23.0%) | 1,196 (67.7%) | 328 (77.7%) | 497 (28.1%) | 154 (36.5%) |
| Bipolar disorder | 66 (24.5%) | 9  (31.3%) | 205 (76.2%) | 36 (69.2%) | 83 (30.9%) | 26 (50.0%) |
| Unipolar depression | 66 (31.3%) | 24 (51.1%) | 122 (57.8%) | 33 (70.2%) | 55 (26.1%) | 14 (29.8%) |
| Personality disorder | 308 (26.9%) | 215 (40.2%) | 732 (63.9%) | 401  (75.0%) | 587 (51.3%) | 349 (65.2%) |
