# Supplementary material for: Mortality, Rehospitalisation and Violent Crime in Forensic Psychiatric Patients Discharged from Hospital: Rates and Risk Factors
Source: PLoS One. 2016 May 19;11(5):e0155906. doi: 10.1371/journal.pone.0155906 (PMC4873227; doi:10.1371/journal.pone.0155906)
Supplement: S2 Table — (DOCX) [file pone.0155906.s002.docx]

**S2 Table.** Rates of adverse outcome by diagnostic group, stratified by comorbid personality disorder (PD)

|  | Death | | Rehospitalisation | | Violent crime | |
| --- | --- | --- | --- | --- | --- | --- |
|  | No PD | PD | No PD | PD | No PD | PD |
| Schizophrenia-spectrum | 456 (21.8%) | 13 (13.1%) | 1,460 (69.9%) | 64 (64.6%) | 613 (29.3%) | 38 (38.4%) |
| Bipolar disorder | 70 (23.3%) | 5 (23.8%) | 224 (74.7%) | 17 (81.0%) | 97 (32.3%) | 12 (57.1%) |
| Unipolar depression | 77 (36.0%) | 13 (29.5%) | 131 (61.2%) | 24 (54.5%) | 56 (26.2%) | 13 (29.5%) |
| Substance use disorder | 456 (49.8%) | 91 (45.7%) | 721 (78.8%) | 157 (78.9%) | 424 (46.3%) | 94 (47.2%) |
